# Emergency Department Attending Physician Variation in Opioid Prescribing in Low Acuity Back Pain

**DOI:** 10.5811/westjem.2017.7.33306

**Published:** 2017-09-18

**Authors:** Jason A. Hoppe, Christopher McStay, Benjamin C. Sun, Roberta Capp

**Affiliations:** * University of Colorado School of Medicine, Department of Emergency Medicine, Aurora, Colorado; † Oregon Health and Science University, Department of Emergency Medicine, Portland, Oregon

## Abstract

**Introduction:**

Despite treatment guidelines suggesting alternatives, as well as evidence of a lack of benefit and evidence of poor long-term outcomes, opioid analgesics are commonly prescribed for back pain from the emergency department (ED). Variability in opioid prescribing suggests a lack of consensus and an opportunity to standardize and improve care. We evaluated the variation in attending emergency physician (EP) opioid prescribing for patients with uncomplicated, low acuity back pain (LABP).

**Methods:**

This retrospective study evaluated the provider-specific proportion of LABP patients discharged from an urban academic ED over a seven-month period with a prescription for opioids. LABP was strictly defined as (1) back pain chief complaint, (2) discharged from ED with no interventions, and (3) predefined discharge diagnosis of back pain. We excluded providers if they had less than 25 LABP patients in the study period. The primary outcome was the physician-specific proportion of LABP patients discharged with an opioid analgesic prescription. We performed a descriptive analysis and then risk standardized prescribing proportion by adjusting for patient and clinical characteristics using hierarchical logistic regression.

**Results:**

During the seven-month study period, 23 EPs treated and discharged at least 25 LABP patients and were included. Eight (34.8%) were female, and six (26.1%) were junior attendings (≤ 5 years after residency graduation). There were 943 LABP patients included in the analysis. Provider-specific proportions ranged from 3.7% to 88.1% (mean 58.4% [SD +/− 22.2]), and we found a 22-fold variation in prescribing proportions. There was a six-fold variation in the adjusted, risk-standardized prescribing proportion with a range from 12.0% to 78.2% [mean 50.4% (SD +/−16.4)].

**Conclusion:**

We found large variability in opioid prescribing practices for LABP that persisted after adjustment for patient and clinical characteristics. Our findings support the need to further standardize and improve adherence to treatment guidelines and evidence suggesting alternatives to opioids.

## INTRODUCTION

Effective pain management is a responsibility of emergency physicians (EP) and an integral part of providing quality healthcare. Recent increased attention to the treatment of pain has contributed to a substantial increase in the prescribing of opioid analgesics in the United States. The U.S. Food and Drug Administration commissioner recently highlighted the critical role medical providers play in the prescription opioid epidemic as deaths continue to rise, contributing to the first decline in American life expectancy since 1993.^1^–^2^

Despite the heightened awareness of harm from opioids and recent interventions, EP opioid-prescribing practices are hypothesized to be highly variable.^3^–^5^ Tamayo-Sarver and colleagues found variation in provider opioid-prescribing choices even when providers were given identical patient scenarios.^6^ High variation suggests lack of provider consensus about how to manage pain and signals opportunities to standardize and improve care. Specifically, reduction of opioid prescribing may reduce the risk of drug diversion and overdose. While some variation is expected because of case-specific issues (e.g., drug allergies, comorbidities), extensive variation is concerning, and identifies the need for system-level interventions to address practice variation in order to increase benefits to ED patients.^7^

Back pain is a model presenting complaint for assessing variations in opioid prescribing. It is one of the most common painful conditions leading to emergency department (ED) visits.^8^ Opioids are commonly used to treat back pain despite the lack of evidence that they are superior to other treatments, with up to 61% of ED patients receive an opioid in the ED or an opioid prescription to treat their pain.^9^–^11^ Further, there is evidence of significant consequences with opioid use for back pain including future opioid use, higher medical costs, and increased disability.^12^–^14^ Furthermore, the American College of Emergency Physicians (ACEP) clinical policy statement on the use of opioids in the ED to treat pain suggests using opioids only when pain is severe, debilitating, or refractory to other treatments.^15^ Similarly the American Academy of Emergency Medicine (AAEM) considers opioids second-line treatment in their clinical practice statement.^16^

To our knowledge, little is known about emergency provider opioid analgesic-prescribing variation and clinical factors associated with this variation in the context of treatment guidelines that suggest non-opioid alternatives. To address this knowledge gap, we examined variation in attending EP prescribing of opioid analgesics to patients with uncomplicated back pain that did not require diagnostic testing or medication treatment in the ED.

## METHODS

### Design

This is a retrospective study evaluating the proportion of adult patients with low acuity back pain (LABP) for whom attending EPs prescribed opioids. The study period was from May 01, 2013, to November 30, 2014. The local institutional review board (IRB) approved this project.

We used strict criteria to identify an ED patient population of similar acuity in order to focus on EP variation rather than patient variation. We limited our study cohort to adult patients (≥ 18 years) with the following characteristics: 1) chief complaint related to back pain symptoms including back injury, back pain, and back/neck/shoulder pain (obtained from a pre-populated pull-down list); 2) discharged home from Intake (see below for description of Intake); and 3) a primary discharge diagnosis of uncomplicated back pain, defined as the Health Care Utilization Project (HCUP) Clinical Classification Software (CCS) number 205 (spondylosis; intervertebral disc disorders; other back problems). HCUP is a federal-state-industry partnership sponsored by the Agency for Healthcare Research and Quality. CCS for *ICD-9-CM* is a diagnosis and procedure categorization scheme based on the *International Classification of Diseases, 9th Revision, Clinical Modification*, and provides a uniform and standardized coding system. CCS collapses *ICD-9* codes into a smaller number of clinically meaningful categories.^17^

**Population Health Research Capsule**

What do we already know about this issue?

Opioids are commonly used to treat back pain in the ED, despite a lack of evidence of superiority to other agents and guidelines recommending against their use.

What was the research question?

How variable are ED attending opioid-prescribing rates within a cohort of patients with comparable acuity?

What was the major finding of the study?

We found a six-fold variation in risk-standardized ED attending opioid-prescribing rates for low acuity back pain.

How does this improve population health?

Extensive variability in opioid prescribing for low back pain suggests the need for interventions to improve guideline adherence and address practice variation.

### Setting

We conducted this study in a single, large academic ED with approximately 100,000 annual ED visits and an admission proportion of 24.9% (12.5% inpatient and 12.4% ED clinical decision unit).

This study focused on patients evaluated in ED Intake. Intake is a front-end physician evaluation model used by our ED. The Intake zone is located by the ED walk-in entrance and is responsible for evaluating all stable patients who arrive by any means other than ambulance. An attending EM board-certified/ eligible physician staffs the area from 9am to 1am daily with two consecutive eight-hour shifts. The average daily Intake census is 75 patients per eight-hour shift (average daily ED census is approximately 277). All patients are evaluated by the EP with three possible destinations: (1) discharge to home (low acuity); (2) Super Track (to be further evaluated and treated by a physician assistant/nurse practitioner for minor imaging, limited studies, minor procedures, or re-evaluation after medications); or (3) to the main ED for additional work-up. Patients discharged from Intake do not have imaging studies, parenteral medications or procedures performed.

Briefly, the Intake process consists of a trained ED tech (EMT-B or paramedic) who greets the patient, obtains vital signs and enters the chief complaint (chosen from a pull-down list). The patient is then placed into one of four Intake rooms, where the attending EP assesses every patient to determine whether or not he/she can be fully evaluated, treated and safely discharged or if they need further work-up and/or treatment in Super Track or the main ED. This approach allows the EP to discharge patients with low acuity conditions (not requiring diagnostic testing or emergent medications) to home after an evaluation. The authors concluded that all patients with back pain discharged directly from Intake without requiring any further work-up were low acuity. We expect this to be a similar population of patients; therefore, we can evaluate the variation of EP treatment decisions independently of patient variation.

Prior to study initiation, an Internet-based statewide prescription drug monitoring program (PDMP) and an institution-specific controlled medication prescription policy existed. The institution-specific policy, implemented in 2012, was not changed during the study. The PDMP in our state was established in 2008 and mandates pharmacist entry for all controlled substance prescriptions at the time the prescription is filled. The PDMP was accessible to all EPs and it remained unchanged with regard to entry of patients and prescriber access during the data collection period. There was no formal policy in the physician practice group or at a state level that structured or directed use of this program. Therefore, physicians used this database at their own discretion with influences on their prescribing patterns unique to each physician.

### Subjects

The physician group includes attending EM board certified/eligible physicians working in the ED. Advanced practice providers (APP) and residents do not work in Intake and therefore were not included. Similar to previous studies, in order to assess the EP opioid prescription variation and increase the confidence in our results, we excluded all providers who evaluated less than 25 patients that met our LABP inclusion criteria.^18^

### Primary outcome

The main outcome of the study was the provider-specific proportion of LABP patients prescribed an opioid analgesic. To determine if there was opioid prescribing variation, for each EP we calculated the percentage of LABP patients prescribed one or more opioid analgesics at ED discharge. We chose receiving a prescription as a binary outcome rather than assessing morphine equivalents because prior work at our institution found that the vast majority of our ED prescriptions are for a small number of pills and similar strength preparations (15 pills, IQR 12–20). Since these are similar to national trends for ED opioid prescribing, small differences between ED opioid prescriptions are unlikely to be clinically relevant.^19^

### Measurements

We extracted LABP ED visits and discharge opioid analgesic prescriptions from Intake from the electronic health record (EHR: Epic 2010 Verona, WI) via computer algorithm. Data collected included EP provider, chief complaint, age, gender, race/ethnicity, insurance status, and opioid prescriptions. No patient identifiers (medical record number or patient identity) were recorded in the database. Race was coded as Black, White, Hispanic or Other. Insurance status was coded as federal (Medicare or Medicaid), commercial, self-pay, medically indigent and other (Worker’s Comp, Veteran’s Affairs, Child Health Plus). We abstracted all medical record data through electronic reports, eliminating potential bias and data entry errors associated with manual abstraction.

We defined an opioid analgesic prescription as any schedule II, III, and IV medications that contained an opioid, including tramadol. We did not include sedatives or stimulants. All prescriptions were ordered electronically via the EHR. We did not evaluate if the patient filled the discharge opioid prescription.

### Analysis

We used descriptive statistics to describe our study population. We compared groups using chi-square analysis for categorical variables and ANOVA for continuous variables. A two-tail p value <0.05 was considered statistically significant. We assessed the association between patient characteristics and receipt of opioid prescription using logistic regression analysis. We reported the odds ratio (OR) and 95% confidence intervals (CI).

We calculated each provider’s percent of LABP patients prescribed an opioid analgesic. Our strict inclusion criteria produced a similar cohort, but we wanted to adjust for patient-related factors that may influence EP opioid prescribing such as age, gender, race, and primary care provider status. It is possible that evaluating and discharging more LABP patients may have an effect on a provider’s proportion of opioid prescribing; to address this we also adjusted for each EP’s back-pain patient volume in Intake. The patient LABP volume consisted of all patients who fit our study inclusion criteria: chief complaint of back pain, final diagnosis of back pain, and were discharged from Intake, with or without an opioid analgesic prescription.

To adjust for patient-related factors, we calculated the risk-standardized opioid prescription proportion at the provider level. The EP risk-standardized opioid prescription proportion was defined as the ratio of observed to predicted number of opioid prescriptions per provider, which was then multiplied by the group’s mean opioid prescription proportion. We used logistic hierarchical regression analysis, where the physicians were considered random effects in the analysis.^20^–^25^ All analyses were conducted in SAS 9.3 (Cary, NC).

## RESULTS

Twenty-three EPs treated and discharged at least 25 LABP patients and were eligible for inclusion in the final analysis; eight (34.8%) were females and six (26.1%) were junior attendings (≤ 5 years after graduation from residency) (Table 1). They treated 943 LABP patients. During the seven-month study period 1,857 patients presented to the ED with a chief complaint related to back symptoms and were discharged from Intake; of these, 1,166 (63%) patients also had a final diagnosis of back pain. We excluded patients seen by providers who had seen less than 25 patients with LABP in Intake, resulting in the final cohort of 943 patients.

Table 2 describes the LABP patients’ characteristics and whether or not they received an opioid prescription. The mean age was 37.8 (SD +/− 12.1); 568 (60.2%) were females, and most patients were minorities, including 289 Blacks (30.7%) and 197 Hispanics (20.9%). The most common insurance coverage was Medicaid (38.0%). When compared with Whites, Blacks were less likely to receive an opioid prescription for LABP (OR 0.65; 95% CI [0.48–0.89]). When compared with patients who were not seen in the ED for back pain in the last 30 days, those who were visiting the ED with a chief complaint of back pain for a second time within 30 days were more likely to receive an opioid prescription on the second visit (OR 1.68; 95% CI [1.03–2.73]). Oxycodone (65%) was the most commonly prescribed opioid in this cohort, followed by hydrocodone (27%) and tramadol (8%).

Figures 1a and 1b show the raw EP opioid prescription variation and the adjusted EP risk-standardized opioid prescription variation, respectively. The unadjusted variation in EP opioid analgesic-prescribing proportion for patients with LABP ranged from 3.7% to 88.1%, a 22-fold variation. The mean unadjusted EP opioid-prescribing proportion was 58.4% (SD +/− 22.2). The adjusted variation in EP opioid-prescribing rates for ED patients with LABP ranged from 12.0% to 78.2%, a 6-fold variation. The adjusted mean EP opioid-prescribing proportion was 50.4% (SD +/−16.4).

## DISCUSSION

In this study, we found a six-fold variation in the provider-specific adjusted proportion of LABP patients prescribed an opioid analgesic. Physician opioid-prescribing practices play an important role in the current opioid epidemic. Wide variability in prescribing decisions for ED discharges has been previously described and, importantly, higher prescribing rates were associated with increased risk of future opioid use.^26^ This study is novel in that we assessed opioid prescribing variability for EP attendings within a homogeneous cohort of patients with a low acuity condition. While there is no accepted “correct” proportion of back pain patients who may benefit from an opioid on discharge, it is reasonable to expect low variability in the setting of national guidelines supporting non-opioid alternatives, lack of evidence of superiority, and evidence of poor long-term outcomes associated with opioids.^8^–^14^ This widespread variation in proportions of opioid prescriptions suggests that ED patients are at risk for both the under-treatment and over-treatment of pain with opioids when presenting to the ED with back pain. This is a major patient safety issue.

Deciding whether or not opioids are the safe and appropriate choice for a given patient is fraught with physician preferences and perceptions. One approach to decreasing overall provider treatment variation is to implement clinical pathways into the ED workflow.^27^–^28^ Clinical pathways help decrease provider practice variation when developed in conjunction with practicing providers and by using evidence-based medicine.^29^–^30^ ED providers are able to access prior controlled medication prescriptions for patients through the use of a PDMP, but many systems are time consuming and there is variability in the interpretation of the information. While the use of the PDMP appears to be the most objective way to identify patients at risk for becoming dependent or even dying from opioid, very little is known about how to best use this critical information in clinical practice.^31^

Another contributing factor to opioid prescription variation relates to the EP’s perception that opioid prescription may be associated with patient satisfaction and the path of least resistance for a rapid discharge. This perception has been contradicted by a recent study suggesting that patient satisfaction scores are not associated with opioid prescription.^32^ Nonetheless, ED providers are asked to rapidly and safely treat pain without the benefit of an established doctor-patient relationship in an environment with limited time and resources. Competing priorities make it difficult to adequately address all of our patients’ needs and questions. Recent shifts in clinical expectations (both administrative and patient specific) without the necessary increased time spent on provider education can put EPs in a difficult position.^33^

This study is the first to describe the EP opioid-prescribing variation in clinical practice within a cohort of patients with comparable acuity. The prescribing information was easily obtained from administrative data and can be easily reproduced in clinical settings where prescribing is done via computer order entry. Using this information to evaluate both random and specific variation in physician practice is an important part of the current healthcare quality environment.^7^ Given the escalation of poor outcomes associated with increased opioid availability,^2^ variation in opioid analgesic prescribing to ED patients requires further study.

Any intervention aimed at decreasing opioid availability and increasing quality care by increasing guideline adherence should include an assessment of doctors’ practice variation, risk tolerance and perceptions. Clinical interventions and policy changes can address opioid-prescribing variation via the use of clinical pathways, embedded decision support, and provider opioid-prescribing metrics. Ultimately, EPs will need to assess the impact of provider variation on patient outcomes with sufficient follow-up and end points. Understanding and evaluating departmental and local hospital variation of prescribing may serve as valuable internal and external benchmarks in the assessment of emergency medicine prescribing safety and quality.

## LIMITATIONS

Our study should be evaluated in the context of a few limitations. First the external validity of our findings is limited because this is a single-center, retrospective study and the use of a process (Intake) that is not universally available in all EDs. While our specific intake process may be somewhat unique, the process of having a provider assessing patients as they present to the ED is not.^32^ Our overall ED patient population and local opioid-prescribing practices may differ from those at other centers; however, the fundamental concept of addressing prescribing variation in the practice of EM remains valid.^35^ To limit our sample we used the combination of chief complaints and final diagnosis, which may have excluded patients with LABP, therefore leading to classification bias. Further, we were unable to track or account for use of our state PDMP in these decisions because of statutory limitations accessing this data. Finally, we did not assess whether patients filled their prescriptions, as we were interested solely in understanding the physician practice habits.

## CONCLUSION

We found significant variation among attending emergency physicians in the decision to prescribe opioids analgesics within a cohort of low acuity back pain patients. This implies a critical need for further assessments of this decision and interventions to promote the safe and effective prescribing of opioid pain medications consistent with national treatment guidelines.

## Figures and Tables

**Figure 1A:**
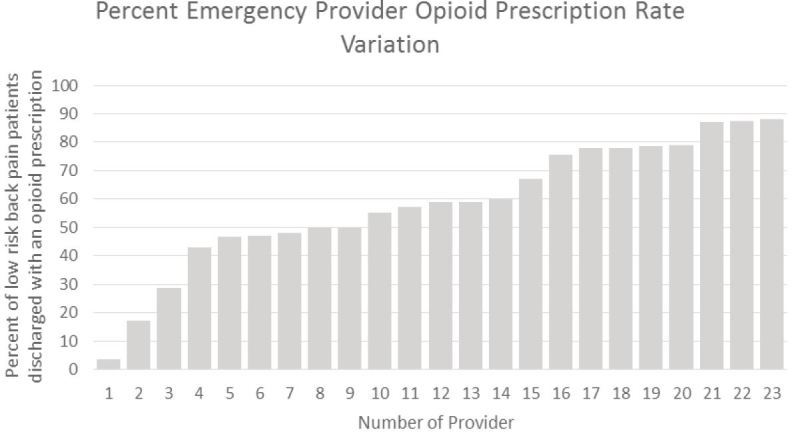
Emergency department attending physician opioid prescribing rates for patients with low acuity back pain.

**Figure 1B:**
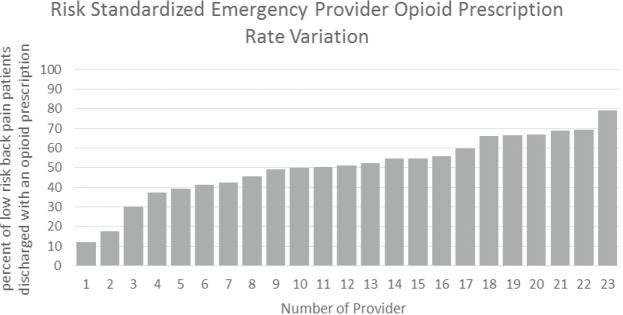
Risk-standardized opioid-prescribing rates of emergency department physicians for patients with low acuity back pain.

**Table 1:** Characteristics of ED attending physicians in a study examining variation in opioid prescribing for low acuity back pain.

Provider characteristics	n=32
Gender	
Male	15 (65%)
Female	8 (35%)
Experience (after residency)	
0–5 years	6 (26%)
>5 years	17 (74%)

**Table 2:** Low acuity back pain patient characteristics.

Patient characteristics	Did not receive opioid N=375 (39.8%)	Received opioid N=568 (60.2%)	Total N=943	Odds of receiving an opioid
Female	292 (51.4%)	276 (57.6%)	568 (60.2%)	1.25 (95%CI 0.96–1.62)
Age (mean)	36.3 (SD 12.2)	38.8 (SD 12)	37.8 (SD 12.1)	1.02 (95%CI 1.01–1.03)
Race				
White	142 (38%)	254 (44.8%)	396 (42.1%)	Reference
Black	133 (35.6%)	156 (27.5%)	289 (30.7%)	0.65 (95%CI 0.48–0.89)
Hispanic	77 (20.6%)	120 (21.2%)	197 (20.9%)	0.87 (95%CI 0.62–1.23)
Other	22 (5.9%)	37 (6.5%)	59 (6.3%)	0.94 (95%CI 0.53–1.66)
Insurance				
Medicaid	144 (38.4%)	214 (37.7%)	358 (38%)	1.09 (95%CI 0.72–1.65)
Medicare	25 (6.7%)	56 (9.9%)	81 (8.6%)	1.65 (95%CI 0.91–2.97)
Private	53 (14.1%)	72 (12.7%)	125 (13.3%)	Reference
Indigent	49 (13.1%)	101 (17.8%)	150 (16%)	1.52 (95%CI 0.93–2.48)
Other	11 (2.9%)	25 (4.4%)	36 (3.8%)	1.67 (95%CI 0.76–3.70)
Self-pay	93 (24.8%)	100 (17.6%)	193 (20.5%)	0.8 (95%CI 0.50–1.25)
Has a PCP				
Yes	161 (42.9%)	274 (48.2%)	435 (46.1%)	1.24 (95%CI 0.95–1.61)
No	214 (57.1%)	294 (51.8%)	508 (53.8%)	Reference
Emergency department visit within last 30 days for back pain	25 (6.7%)	61 (10.7%)	86 (9.1%)	1.68 (95%CI 1.03–2.73)

*PCP,* primary care physician.
